# Myositis Ossificans of the Masseter Muscle: A Rare Post-traumatic Case

**DOI:** 10.7759/cureus.97011

**Published:** 2025-11-16

**Authors:** Charbel Daoud, Karl El Mendelek, Lynn Daoud, Jihad Khoury

**Affiliations:** 1 Department of Otolaryngology, Head and Neck Surgery, University of Balamand, Beirut, LBN; 2 Department of Otolaryngology, Head and Neck Surgery, Saint George Hospital University Medical Center, Beirut, LBN; 3 Gilbert and Rose-Marie Chagoury School of Medicine, Lebanese American University, Byblos, LBN

**Keywords:** heterotopic ossification, masseter muscle, masticatory muscles, myositis ossificans, myositis ossificans traumatica, trismus

## Abstract

Post-traumatic myositis ossificans of the masseter is an uncommon cause of severe trismus without a standardized management pathway. A 23-year-old man developed progressive jaw stiffness six months after facial trauma, with maximal interincisal opening <10 mm. Imaging demonstrated mature heterotopic ossification bridging the mandibular ramus and zygomatic arch. Excision of the ossified masseter was performed via a combined intraoral and external approach. Trismus recurred within three months, and re-operation through the prior incision removed residual ossification with coronoidectomy. No adjuvant therapy was used; supervised jaw-opening physiotherapy was instituted. Early postoperative improvement was observed, and at approximately two months after the second procedure, mouth opening reached about three finger breadths with ongoing exercises and no clinical evidence of recurrence. This case underscores that timely recognition of masseteric myositis ossificans and tailored stepwise management, including coronoidectomy when indicated, can restore function and meaningfully improve quality of life.

## Introduction

Myositis ossificans (MO) is defined as the heterotopic bone formation within skeletal muscle, most often after blunt injury or repetitive microtrauma [1,2]. It is a benign, self-limiting process that typically manifests as a solitary intramuscular lesion [3]. MO is classically divided into two entities: myositis ossificans progressiva (MOP) and myositis ossificans traumatica (MOT). MOP, also termed fibrodysplasia ossificans progressiva (FOP), is an autosomal-dominant disorder featuring early-onset, progressive extraskeletal ossification that begins in childhood with potential respiratory compromise and severe disability [4]. In contrast, MOT follows direct trauma or repetitive micro-injury and is thought to arise from aberrant osteogenic differentiation during soft-tissue repair [3]. Although MOT most often affects limb muscles, head and neck involvement is uncommon, and masseter muscle involvement is particularly rare [4,5]. Because progressive trismus after facial trauma can rapidly compromise oral function, early recognition of masseter involvement is important to enable timely jaw-opening physiotherapy and close monitoring before severe fibrotic restriction develops. This article presents a case of masseteric MO, highlighting the clinical presentation, diagnostic evaluation, operative management, and outcomes.

## Case presentation

**Figure 1:**
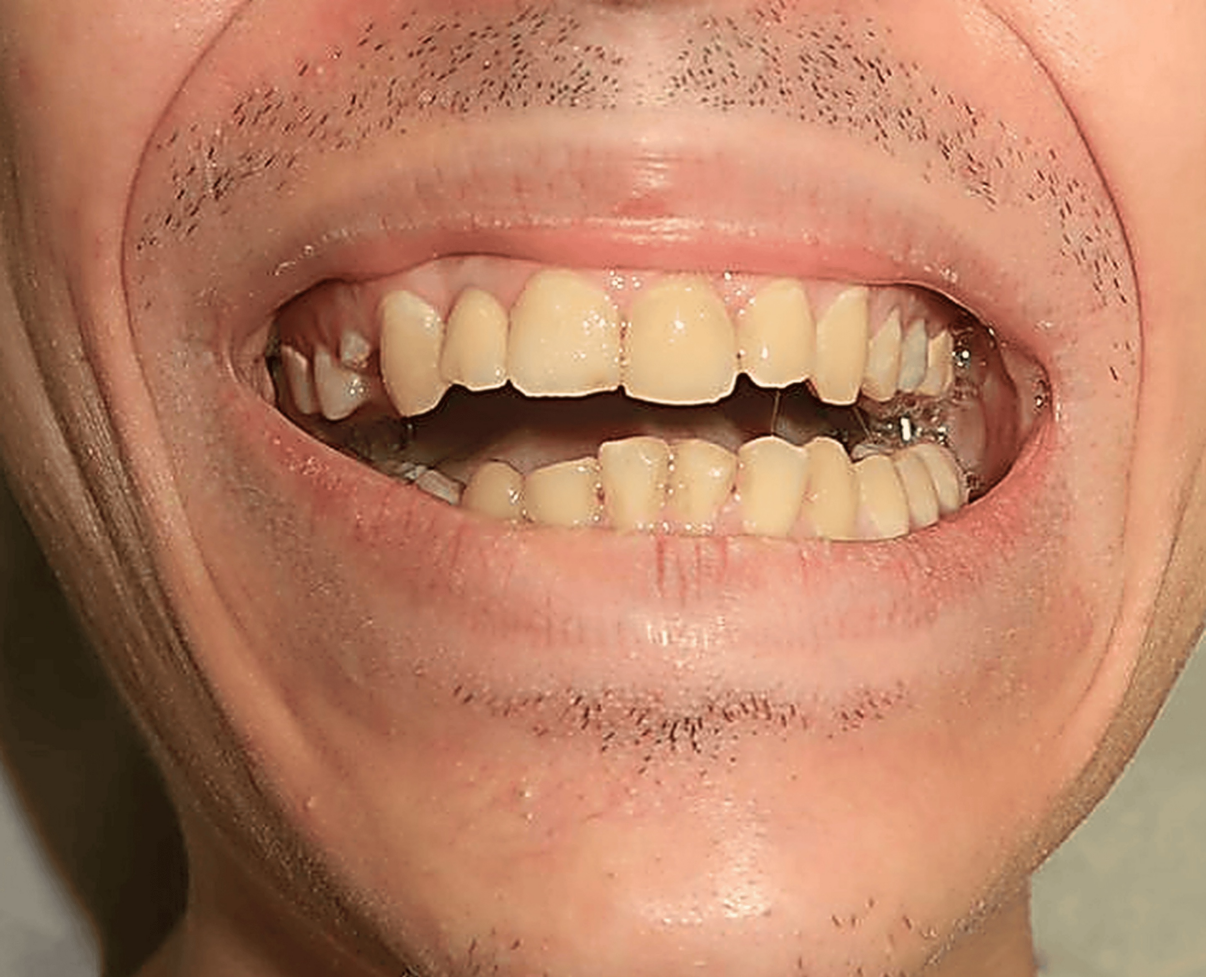
Preoperative clinical photograph demonstrating restricted mouth opening (maximal interincisal distance <10 mm).

A 23-year-old male patient with no prior medical history presented six months after a motor vehicle accident with left facial trauma (deep cheek laceration and comminuted zygomatic fracture treated by open reduction and internal fixation at an outside hospital). Initial postoperative recovery after fracture fixation was uncomplicated; the patient received routine instructions for basic jaw-opening physiotherapy using tongue depressors (approximately 2-3 sessions per day) and attended standard early follow-up visits without significant trismus or suspicion of MO. Over the subsequent months, however, he developed progressive jaw restriction culminating in near-complete jaw locking. On presentation, maximal interincisal opening was less than 10 mm, with left-sided edema and pain, while the facial nerve was clinically intact (Figure 1).

**Figure 2:**
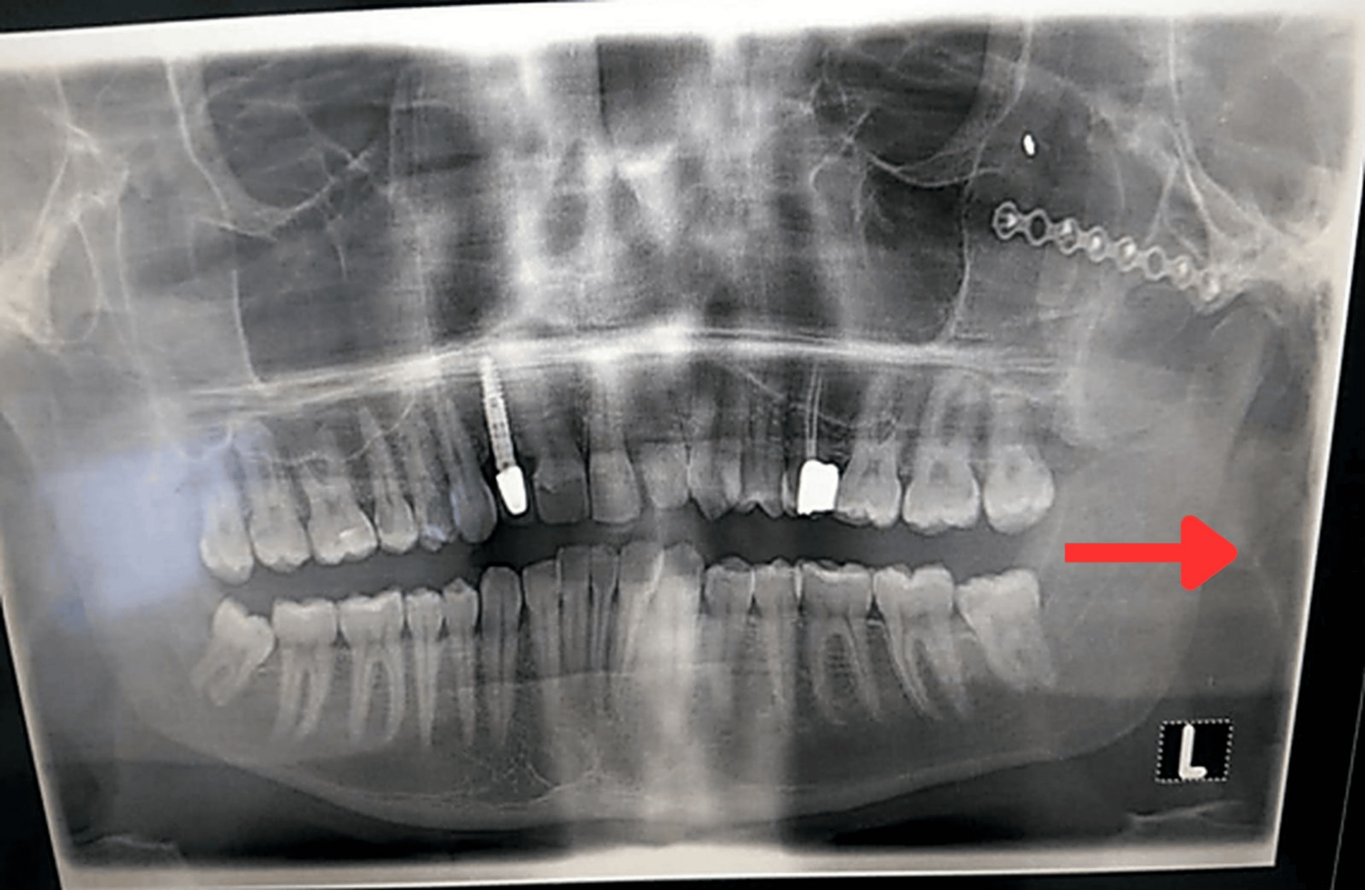
Panoramic radiograph at presentation demonstrating left zygomatic fixation hardware and heterotopic bone bridging the left mandibular ramus and zygomatic arch (red arrow), consistent with masseteric myositis ossificans.

**Figure 3:**
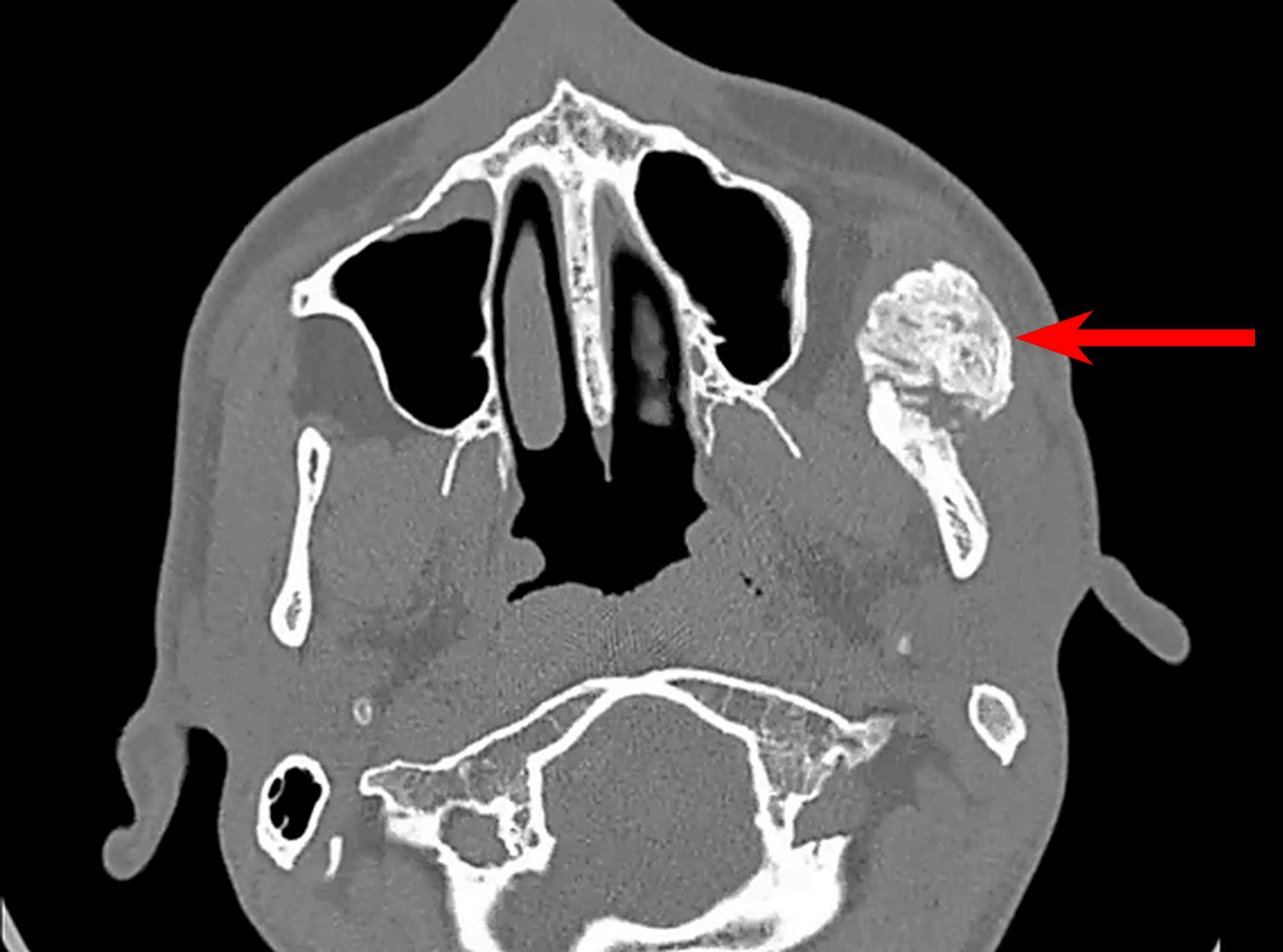
Axial CT (bone window) demonstrating heterotopic ossification of the left masseter (red arrow).

**Figure 4:**
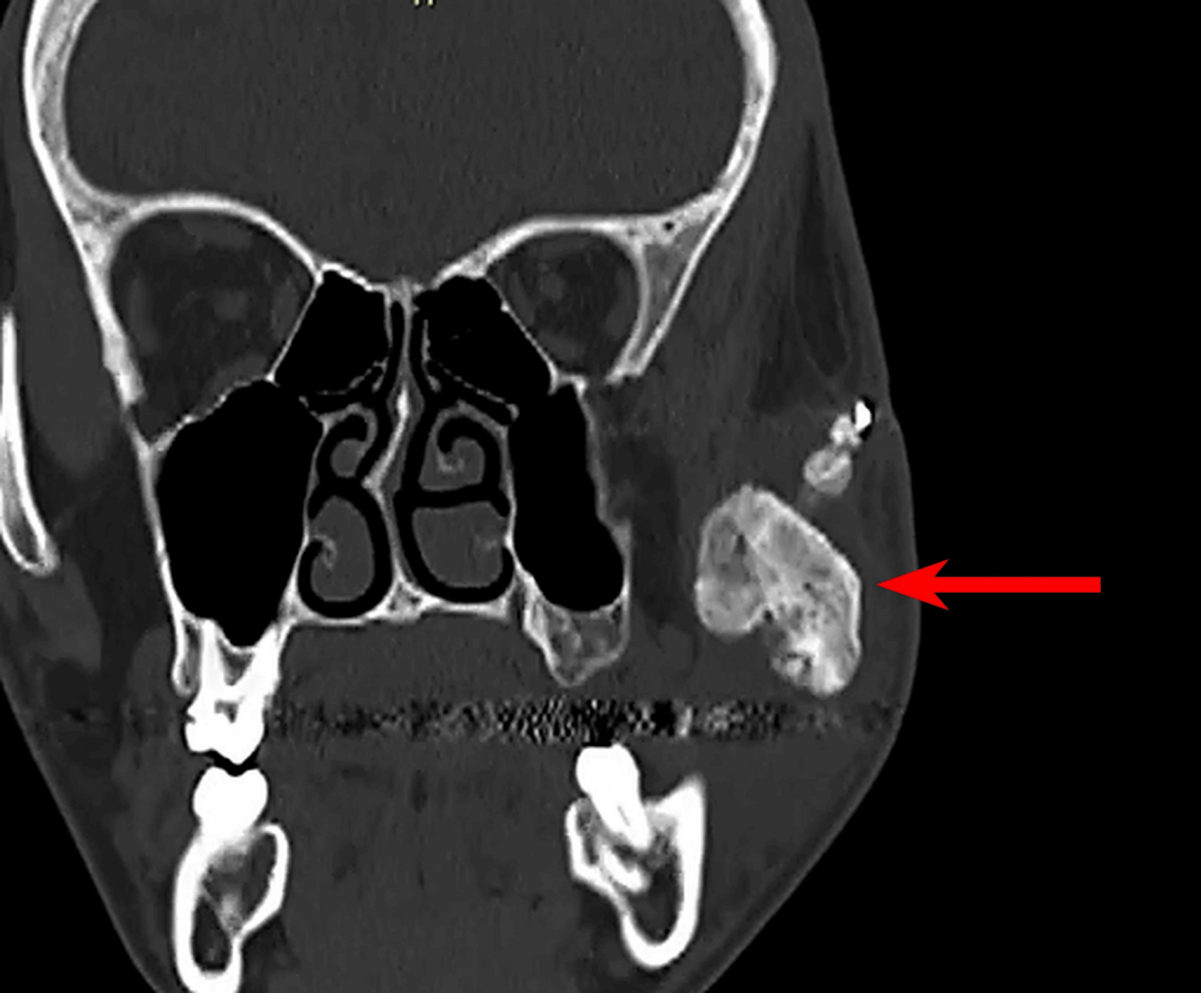
Coronal CT (bone window) showing heterotopic ossification in the left masseter muscle (red arrow).

**Figure 5:**
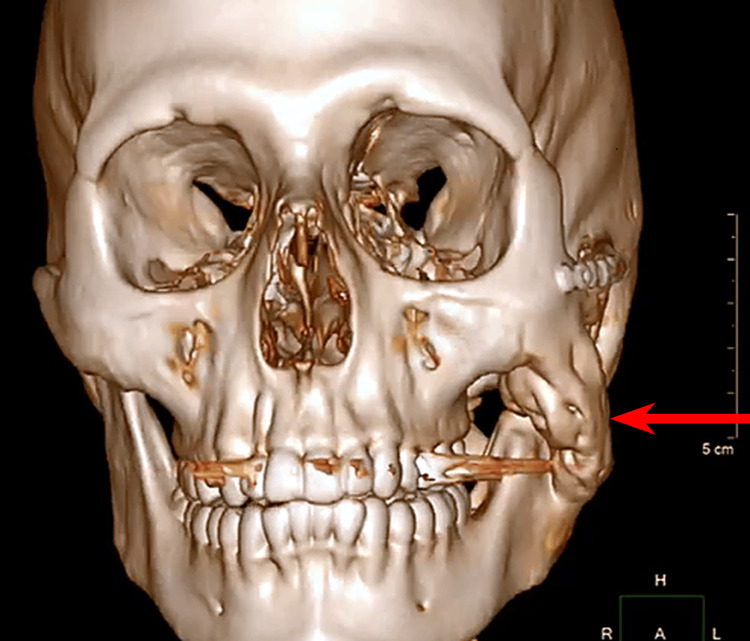
3D CT reconstruction (frontal view) showing heterotopic ossification in the left masseter muscle (red arrow).

Panoramic radiography and computed tomography scans demonstrated mature heterotopic ossification bridging the left mandibular ramus to the zygomatic arch, consistent with MO of the masseter muscle (Figures 2-5). 

Considering the patient's progressive trismus, persistent pain, and imaging findings confirming MO, operative management via a dual approach was planned to resect the ossified mass and restore jaw function.

**Figure 6:**
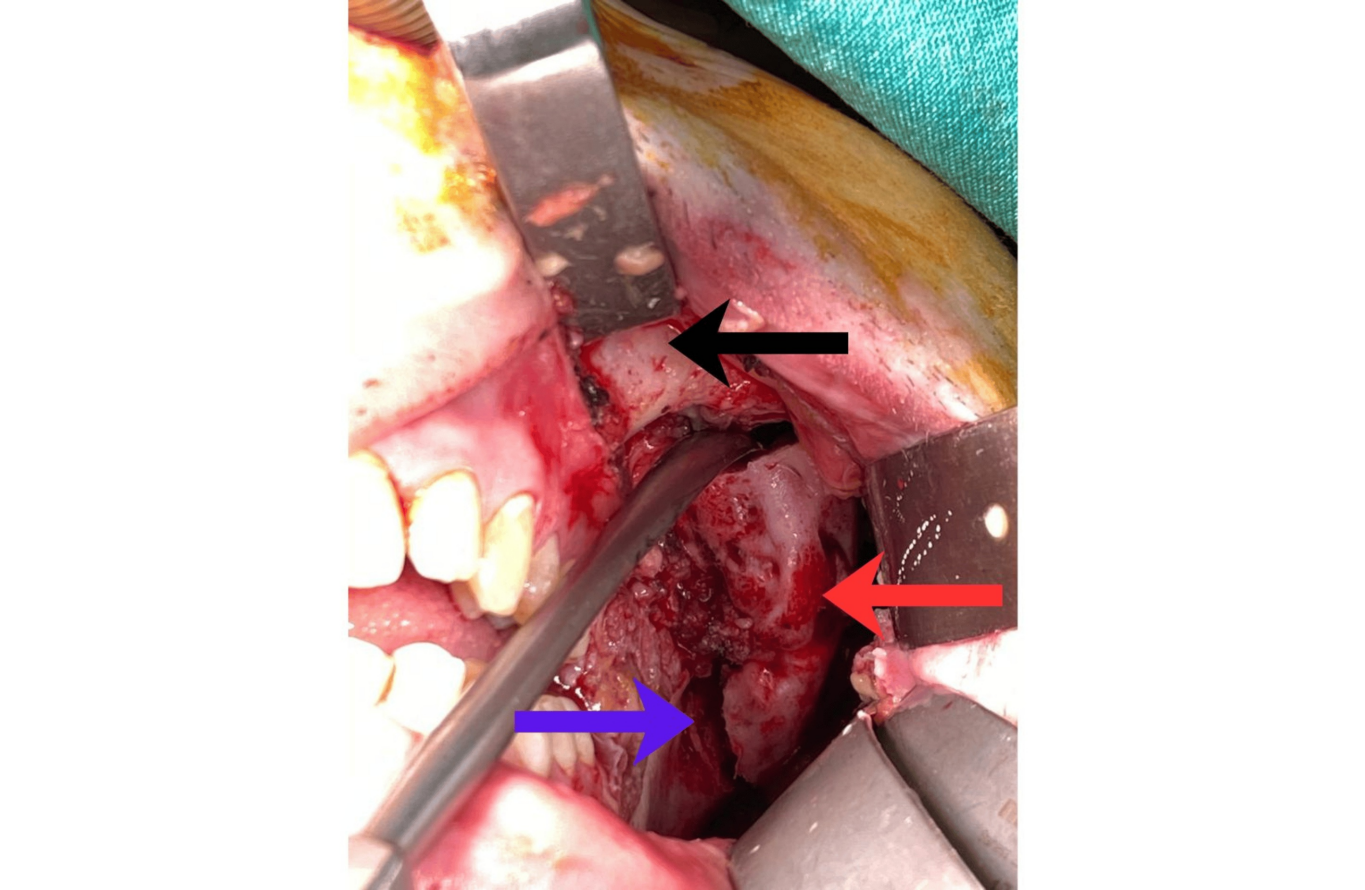
Intraoperative transoral exposure of the ossified left masseter (red arrow) bridging the mandibular ramus (blue arrow) and zygomatic arch (black arrow).

**Figure 7:**
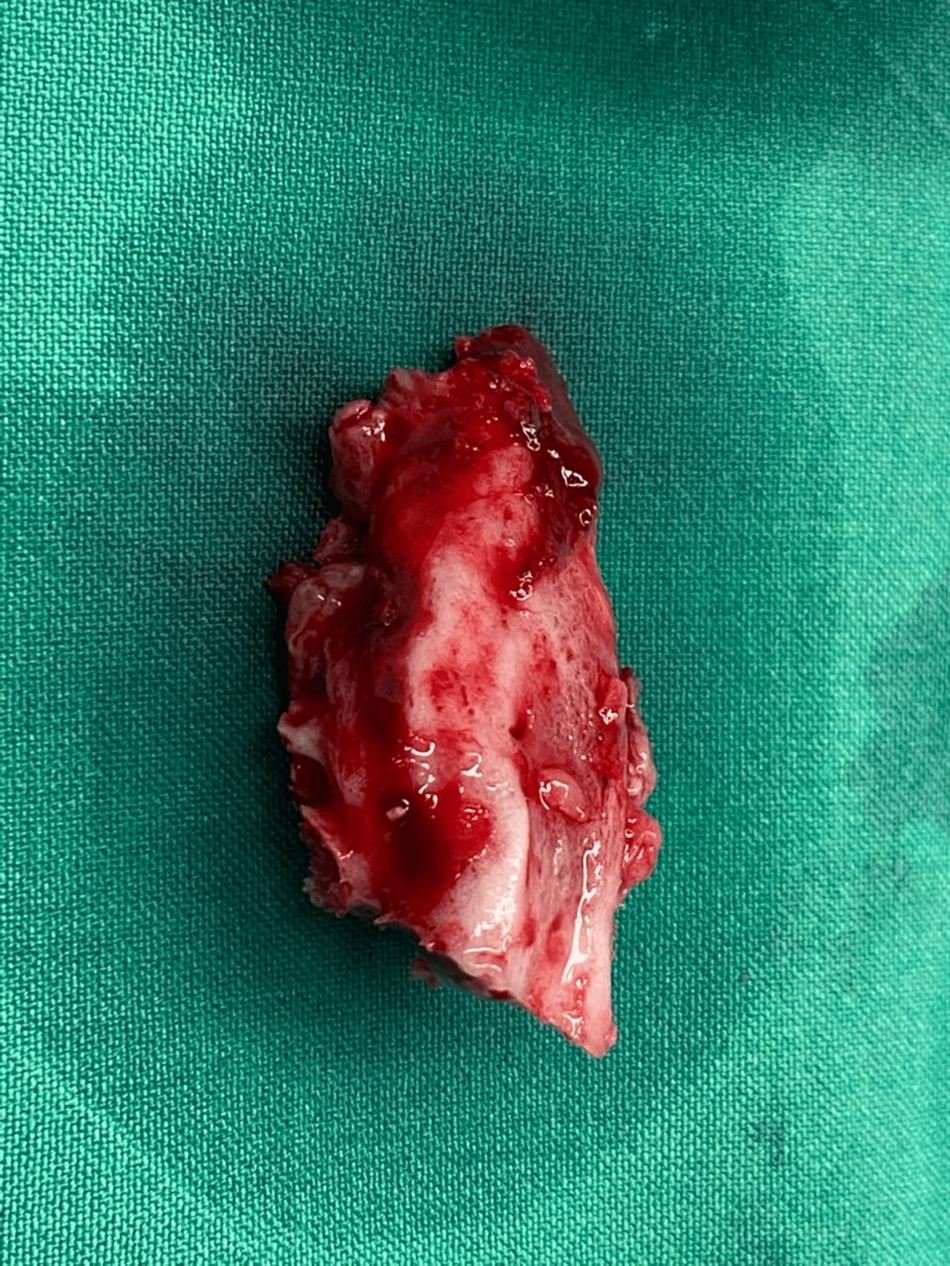
Gross resected specimen.

Surgical removal of the lesion was performed under general anesthesia through a combined intraoral and external approach. The lesion was exposed and released from its mandibular (angle/ramus) and zygomatic attachments, taking care to preserve the facial nerve branches as well as the Stensen’s duct (Figure 6). The specimen was removed en bloc, and bony margins were smoothed and polished (Figure 7). The pre-existing osteosynthetic plate and screws were removed. Maximal passive mouth opening reached 35 mm intraoperatively.

**Figure 8:**
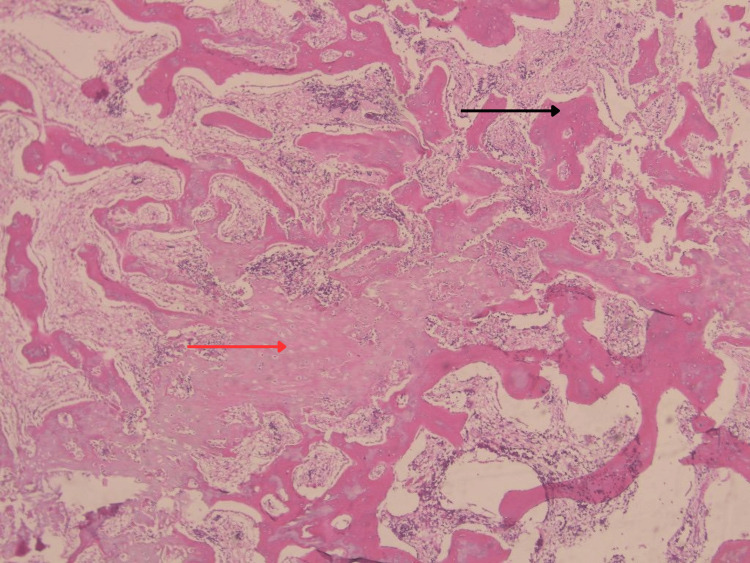
Histopathological examination (H&E) of masseteric myositis ossificans. Irregular trabeculae of woven lamellar bone within a fibrovascular stroma (black arrow) and peripheral areas of unmineralized osteoid matrix (red arrow) demonstrate the characteristic zonal maturation pattern.

On postoperative day 1, mouth opening measured approximately 22 mm, and supervised jaw-opening physiotherapy was initiated. Histopathology showed mature lamellar bone with a zonal maturation pattern, consistent with MO (Figure 8).

Within the first postoperative trimester, the patient developed progressive recurrent trismus. Imaging showed re-ossification in the masseter/coronoid region. One year later, recurrence was identified, and a second operation was performed. Through the prior external incision, dissection proceeded to the coronoid process; the ossified segment was excised along with a coronoidectomy. The previously resected zygomatic arch was left unchanged, and the mandibular cortex was not violated. Adequate intraoperative mouth opening was restored.

At early follow-up, two months postoperatively, the patient achieved a three-finger breadth opening with ongoing physiotherapy (supervised jaw-opening exercises 5-6 times/day using a progressive protocol for 6-8 weeks). At the latest follow-up, six months after the second surgery, this opening remained stable on continued home exercises, with no clinical evidence of recurrent ossification. The evolution of maximal interincisal opening and recurrence status over the course of management is summarized in Table 1.

**Table 1:** Evolution of maximal interincisal opening and recurrence status.

Time point	Clinical context	Maximal interincisal opening	Recurrence/imaging findings
Presentation to our clinic (six months after initial trauma and fracture fixation)	Progressive trismus with near-complete jaw locking	<10 mm	Imaging: mature heterotopic ossification bridging the left mandibular ramus and zygomatic arch
Postoperative day 1 after the first surgery	Early postoperative improvement	≈35 mm	No residual bridging reported clinically
Within the first postoperative trimester after the first surgery	Progressive recurrent trismus	<20 mm	Imaging: re-ossification in the left masseter/coronoid region
Two months after the second surgery (≈ one year after recurrence)	After repeated excision and coronoidectomy, on physiotherapy	Three-finger breadth opening (clinically)	No clinical evidence of recurrent ossification at this stage
Six months after the second surgery (latest follow-up)	Stable function on continued home jaw exercises	Three-finger breadth opening (maintained clinically)	No clinical evidence of recurrent ossification

## Discussion

Although uncommon, MO of the masseter muscle can significantly impair a patient's quality of life and functional abilities due to the resulting trismus and restricted jaw mobility. Trauma is the leading cause, accounting for 60%-75% of cases [6,7]. The most common symptom is progressive limitation of mouth opening, as seen in our case [8].

When MO affects masticatory muscles, the masseter is the most involved one [9], then comes the medial pterygoid, lateral pterygoid, and finally the temporalis muscle [8]. The increased occurrence of MO in the masseter muscle may be due to its external location, making it more susceptible to direct trauma [5,8,9].

The largest series of cases has demonstrated a higher incidence in males, with a ratio of 2.5:1 [5], with presentation typically in the third to fifth decades [5,8]. In the progression of MO, three overlapping stages of evolution are commonly described: early, intermediate, and mature. During the early stage, which occurs within the first four weeks following injury, an inflammatory response precedes ossification, and therefore, calcifications are not usually visible on radiographs. The lesion enters the intermediate stage between four to eight weeks, where calcification becomes radiographically evident. The mature stage is marked by pronounced peripheral bone formation, with the lesion continuing to mature over the following months until it consolidates [3]. In our case, the patient was already in the mature stage of the disease. Earlier cross-sectional imaging during the period of progressive trismus might have identified the ossification stage sooner and prompted earlier optimization of jaw physiotherapy and closer monitoring, although whether this would have altered the need for surgery is uncertain.

Importantly, MO can occur in the absence of a clear history of trauma, as demonstrated in a case report of pseudomalignant MO involving multiple masticatory muscles [1]. Therefore, reliance on imaging and, in some cases, histopathology is essential to establish the correct diagnosis. The radiographic appearance of MO can closely mimic other conditions affecting the muscles of mastication, such as malignant soft tissue tumors, rhabdomyosarcoma, or aggressive fibromatosis [9]. Therefore, distinguishing MO from these other entities is critical, as the appropriate management strategies differ significantly. Radiographic findings, including panoramic radiography and CT, are crucial for diagnosis, as they can demonstrate the characteristic heterotopic ossification within the muscle [3,10]. In the early stages of the disease, standard radiographs may be normal, whereas in more advanced stages they typically show a peripheral rim of ossification surrounding a relatively radiolucent center [3,10]. CT is more sensitive than radiography and is generally considered the imaging modality of choice, as it clearly depicts the zonal pattern of mineralization and the relationship of the lesion to adjacent bone [3,10]. In this patient, CT was, therefore, obtained after panoramic radiography suggested a dense radiopaque mass, and it confirmed a fully mineralized bony bridge between the mandibular ramus and zygomatic arch, which was critical for operative planning. MRI findings depend on the stage of the lesion: early or intermediate lesions show a heterogeneous T2-hyperintense mass with edematous or fibrovascular tissue and only partial mineralization, whereas more mature lesions demonstrate a hypointense peripheral rim on T1- and T2-weighted images corresponding to cortical bone [10,11]. MRI is therefore most helpful when the diagnosis is uncertain or when distinguishing active inflammatory tissue from both mature ossification and malignant soft-tissue mimics is likely to alter management [10,11]. Because CT already demonstrated a typical mature bony bridge in our patient, an additional MRI was not obtained, as it was unlikely to change staging or surgical strategy, and the overall imaging appearance was consistent with a mature stage of MO.

The ossification pattern and histopathological features also help distinguish MO from other etiologies. The hallmark feature of MO is the formation of mature, lamellar bone within the muscle tissue [12]. This process typically begins at the lesion's periphery and progresses inward, creating a distinct zonal pattern [13]. The histopathology of MO evolves through three main stages. In the early stage, the lesion is highly cellular with immature mesenchymal cells that appear benign. The lesion develops cartilage and immature bone during the intermediate stage, both of which appear benign. By the mature stage, the lesion consists of mature lamellar bone [3].

Due to the limited data and the small number of reported cases, there are no clear recommendations for the treatment of MO, particularly when it affects the masseter muscle. However, most authors agree that surgery is generally the preferred approach as the evidence regarding the effectiveness of conservative management is inconclusive. The optimal timing for surgery also remains debatable due to insufficient data. Some authors advocate for immediate surgical intervention upon diagnosis, while others recommend waiting for 6 to 12 months to allow the lesion to mature and fully calcify before proceeding with the surgery [5]. Although it was previously thought that early surgery might increase the risk of recurrence, more recent studies suggest that early excision has minimal risk and that the decision should consider the etiology of MO rather than timing alone [3]. In our situation, the patient presented 6 months post-trauma; therefore, the lesion's natural progression already determined the decision on surgical timing.

In addition, some authors advocate for the interposition of graft substances, like abdominal or buccal fat pads, to prevent hematoma formation, collapse, and relapse after excision [5,9]. The use of suction drains in the resection gap also helps prevent hematomas and recurrence [9].

Treatment options following surgery for MO encompass etidronate disodium, which inhibits crystal growth and calcification but carries a risk of osteomalacia; nonsteroidal anti-inflammatory drugs to prevent pre-osteoblast differentiation; low-dose radiation to inhibit the transformation of mesenchymal cells into osteoblasts; ascorbic acid to reduce procollagen type III synthesis; steroid injections for which there is still limited evidence; and warfarin, which reduces the production of osteocalcin [5,9]. However, evidence for these adjuvant modalities remains limited, and none has been established as standard of care. In our patient, we therefore pursued surgery without interpositional material or adjuvant pharmacologic or radiotherapy.

Beyond the choice of adjuvant therapy, operative planning must also address facial nerve preservation. This is a key concern in masseteric MOT because the ossified mass often lies close to, or partially envelops, branches of the facial nerve. Fité-Trepat et al. described a masseteric MO in which the marginal mandibular branch was completely encased by ossified tissue and had to be sacrificed, resulting in postoperative paralysis [5], whereas Boffano et al. reported successful excision of medial pterygoid MO using combined intraoral and preauricular/submandibular approaches without permanent facial nerve deficit [8]. Drawing on these reports and our own experience, several intraoperative strategies appear helpful: planning external incisions to allow dissection in a sub-superficial musculoskeletal system (sub-SMAS) plane, reusing previous scars when feasible, limiting lateral dissection to the ossified mass, and combining transoral with targeted extraoral exposure so that facial nerve branches are identified and gently mobilized rather than stretched or cauterized blindly. In our case, the initial dual approach permitted complete removal of the ossified mass and hardware while protecting facial nerve branches. Despite early improvement, recurrent trismus with coronoid-related mechanical limitation developed and was effectively managed by re-operation with coronoidectomy and excision of the recurrent ossification. In the broader literature, coronoidectomy has been used selectively when imaging or intraoperative findings demonstrate coronoid or temporomandibular joint involvement or when residual bony contact limits opening after excision of the heterotopic mass [4,8,9]. Boffano et al. reported that in their series of masticatory muscle MO, most patients were treated with excision of the ossified muscle alone, with adjunctive coronoidectomy or condylectomy reserved for a minority of cases [8]. Reports of masseteric MO likewise describe coronoidectomy only when the coronoid process participates in the ossified bridge [4,5,7,9]. This two-stage course, therefore, supports an individualized approach in which arch-side exposure is valuable at index surgery for safe, thorough resection, and coronoidectomy is added when recurrence or persistent restriction suggests coronoid involvement.

Structured postoperative physiotherapy was instituted to maintain gains, consisting of supervised jaw-opening exercises 5-6 times per day using a progressive stretching protocol over at least 6-8 weeks, followed by continued home exercises. Although no standardized regimen has been validated, published cases of masticatory MO similarly emphasize early, intensive active and passive mouth-opening exercises after surgery, often described as intensive jaw physical therapy, to consolidate improvements and reduce the risk of fibrotic relapse [7,8,12]. Early follow-up of our patient shows functional improvement with a stable three-finger breadth opening.

This report has the limitations inherent to a single case: functional assessment was largely based on maximal interincisal opening rather than standardized pain or patient-reported outcome measures, and follow-up, although extended to six months after the second surgery, does not yet allow firm conclusions about very long-term recurrence.

## Conclusions

Masseteric MO is an uncommon but disabling sequel of facial trauma that should be considered in any patient with progressive trismus, particularly when symptoms worsen after an initial period of recovery. Accurate staging through imaging and confirmation by histopathology guides the timing and extent of surgery. In our patient, an initial dual-approach resection improved jaw opening, while recurrence with coronoid involvement required re-operation with coronoidectomy. Structured physiotherapy then yielded a stable three-finger breadth opening at two months, which was maintained at six months without interpositional grafts or adjuvant therapies. This case underscores that prompt recognition of masseter involvement and early institution of jaw-opening exercises may help limit severe fibrotic restriction while definitive surgical planning is undertaken.

In the absence of robust data for pharmacologic or radiotherapy adjuvants, a pragmatic pathway (maturation-guided resection, consideration of coronoid involvement, early rehabilitation, and close surveillance) offers a safe, reproducible strategy, although longer-term follow-up is still needed to confirm durability beyond six months.
